# Secondary SUNCT syndrome caused by dorsolateral medullary infarction

**DOI:** 10.1186/s10194-016-0604-2

**Published:** 2016-02-17

**Authors:** Di Jin, Ya-Jun Lian, Hai-Feng Zhang

**Affiliations:** Department of Neurology, the First Affiliated Hospital, Zhengzhou University, 1 Jianshe East R, Zhengzhou, 450052 Henan Province People’s Republic of China

**Keywords:** Dorsolateral medullary infarction, SUNCT syndrome

## Abstract

Short-lasting unilateral neuralgiform headaches with conjunctival injection and tearing (SUNCT) is a rare headache syndrome which belongs to trigeminal autonomic cephalalgias. Though the majority of SUNCT syndrome is idiopathic, more and more cases of secondary SUNCT syndrome have been reported recently. In this study, we present a case of symptomatic SUNCT syndrome caused by acute dorsolateral medullary infarction which was verified by brain MRI(magnetic resonance imaging). Up to now, there is not absolutely effective treatment for SUNCT syndrome. However, in our case, SUNCT was completely resolved after conventional treatment for cerebral infarction without specific drug intervention.

## Background

Short-lasting unilateral neuralgiform headaches with conjunctival injection and tearing(SUNCT) is a relatively rare headache disorder. It is characterized by unilateral paroxysmal headache accompanied by autonomic manifestations such as conjunctival injection, lacrimation, nasal stuffiness and rhinorrhoea. In the vast majority of cases the aetiology is unknown until a case of symptomatic SUNCT syndrome was first proposed by Sjaastad O in 1989 [1]. Since then, increasing identifiable lesions have been reported. Herein we describe a case of SUNCT syndrome occurring shortly after the onset of dorsolateral medullary infarction. Similar case was reported by Penart in 2001 [2], but evidence to prove the relationship between acute infarction and SUNCT was insufficient.

## Case presentation

**Fig. 1:**
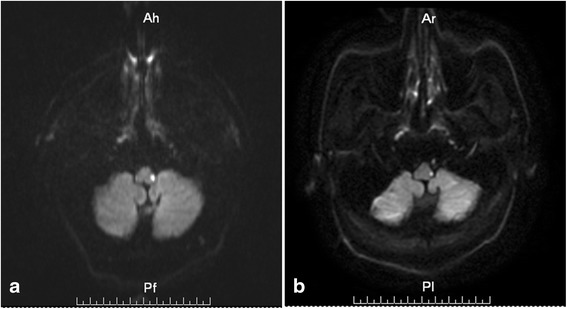
MR dffusion weighted imaging of infarct lesion in the left side of dorsolateral medulla. **a** DWI shows the left-sided dorsolateral medullary infarction. **b** The infarct volume became smaller at the ninth day after admission to our hospital

A 64-year-old male with a remote history of hypertension and diabetes developed acute onset ataxia, nausea and vertigo without obvious inducement on July 26th, 2015. All symptoms were maximal at onset. After one day, left ptosis with miosis commenced and a sensory disturbance affecting the peripheral region of the left face developed. He received treatment for acute cerebral infarction at the local hospital, included aspirin 100 mg qn, atorvastatin calcium 20 mg qn, and some liquid which were used to improve cerebral circulation and so on. After several days of intravenous infusion therapy, there was no obvious improvement, then he transferred to our hospital on August 5th, 2015. Neurological examination confirmed a left Horner’s syndrome, hypoesthesia of the peripheral region of left face, and poor tandem gait. Biochemical investigation and autoimmune related indicators were within normal limits. Brain MRI (magnetic resonance imaging) revealed a new infarct lesion in the left side of dorsolateral medulla (Fig. 1a). Some old lacunar infarct in bilateral basal ganglia and demyelination lesions in right frontal lobe were also found. MRA(magnetic resonance angiography) showed occlusion of the left vertebral artery.

Paroxysmal left-sided pain associated with ipsilateral dacryorrhea, conjunctival injection, rhinorrhoea and flushing on the forehead started on August 8th , 13 days after the onset of the initial symptoms. The headache was stabbing in nature and localized around the left eye with a brief duration, typically from 3 to 10 s. Headache frequency was one to four per day. Triggers couldn’t be found out. Attacks can occur at any time throughout the day, mainly during the daytime. The detailed description of the headache and associated ipsilateral autonomic manifestations fulfilled the key diagnostic features of SUNCT [3]. Then secondary SUNCT syndrome caused by dorsolateral medullary infarction was diagnosed as the consistency in space and time. Conventional treatment for cerebral infarction was undertaken without specific drug intervention for the headache. Then we found the infarct volume became smaller when we rechecked brain DWI on August 14th (Fig. 1b). The headache and Horner’s syndrome resolved on August 27th, while only slight ataxia and hypoesthesia leaved. So far, the headache has disappeared for five months.

## Discussion

The temporal relationship between the symptoms of acute cerebral infarction and subsequent SUNCT suggests that these events were linked in our patient. Partial improvement of medullary infarction and complete resolution of headaches after conventional treatment for cerebral infarction also support this hypothesis.

Actually, the coexistence of SUNCT syndrome and presumed brainstem infarction had been reported by Penart in 2001 [2]. A 63-year-old male presented with acute onset nausa, ataxia, vertigo and a sensory disturbance affecting the left side of the face and right lower quadrant. After seven days, he experienced symptoms presented with severe unilateral left-sided temporal pain lasting about 20 s to 3 min, to make matters worse, left ptosis with miosis commenced. The boots were stabbing in nature, associated with left-sided redness of eye, lacrimation and nasal congestion. The maximum headache frequency was about 8 per day. This case fulfilled diagnostic criteria for SUNCT. Brain MRI and MRA were applied, revealed signs of an old left cerebellar infarct and an occluded left vertebral artery without evidence of new infarct lesions. Paroxsymal headache had improved after several weeks without specific treatment. The Horner’s syndrome and symptoms of SUNCT resolved over one month. Compared with our case, it lacked imaging evidence of new brainstem infarction. However, headaches in both cases were resolved after several weeks with no specific drugs such as gabapentin, topamax or lamotrigine were used.

We have summarized the previous reports and concluded that most of secondary SUNCT are associated with posterior fossa abnormalities. For example, epidermoid tumor of the cerebello-pontine angle [4], vertebral basilar artery malformation [5], cavernous hemangioma of the brainstem [6] and vascular malformation of the cerebello-pontine angle [7], all of the above diseases can induce secondary SUNCT syndrome. Complete remission may be achieved if we remove the intracranial potential risk factors. For instance, the severe pain had been cured by surgical treatment in the case of vascular malformation of the cerebello-pontine angle [7]. According to many researchers, mechanical stimulation of the leasions to trigeminal root may be the reason [8]. Actually, the pathophysiology of SUNCT is ambiguous up to now. The features of SUNCT syndrome are thought to be secondary to the activation of trigeminal-autonomic reflex involving the trigeminal nerve and facial parasympathetic outflow, while the irritation of the trigeminus root could explain the pain [5]. In our case, the ischemic lesion located in medulla could be responsable for the clinical manifestation because of its involvment of the trigeminal system.

But recently more and more secondary SUNCT syndrome is associated with the acute onset of some diseases, improvement of these diseases may induce complete resolution of symptoms of SUNCT. A case of SUNCT syndrome occurring shortly after the onset of neuromyelitis optica was reported by Kursun in 2006, and headache with autonomic features had improved after one week treatment of steroid [9]. Several years later, a case of SUNCT in a man with progressive relapsing multiple sclerosis(MS) was reported [10]. Attacks of MS was consistent with separate episoded of SUNCT, and the short-lasting headache resolved spontaneously when entering remission stage of MS. Interestingly, both of the above diseases were found lesions affected the brainstem. Then a case of secondary SUNCT syndrome following acute sphenoiditis was presented by Pong in 2013, after a two-week course of amoxicillin-clavulanatev, the patient finally had complete resolution of her symptoms [11]. The specific pathogenesis remains to be further discussed.

## Conclusion

Secondary SUNCT syndrome is rarely caused by dorsolateral medullary infarction. We should acquaint ourselves with the possible reasons in order to decrease the missed diagnosis or misdiagnosis rate of secondary SUNCT syndrome.

## Consent

Written informed consent was obtained from the patient for publication of this Case report and any accompanying images. A copy of the written consent is available for review by the Editor-in-Chief of this journal

## Abbreviations

DWI: Diffusion weighted imaging
MRI: Magnetic resonance imaging
MS: Multiple sclerosis
SUNCT: Short-lasting unilateral neuralgiform headaches with conjunctival injection and tearing
